# Readiness assessment for pragmatic trials (RAPT): a model to assess the readiness of an intervention for testing in a pragmatic trial

**DOI:** 10.1186/s12874-019-0794-9

**Published:** 2019-07-18

**Authors:** Rosa R. Baier, Eric Jutkowitz, Susan L. Mitchell, Ellen McCreedy, Vincent Mor

**Affiliations:** 1 Center for Long-Term Care Quality & Innovation, Brown University School of Public Health, Box G-S121-6, 121 South Main Street, Providence, RI 02912 USA; 2 Department of Health Services, Policy & Practice, Brown University School of Public Health, Box G-S121-6, 121 South Main Street, Providence, RI 02912 USA; 3 Center for Gerontology & Healthcare Practice, Brown University School of Public Health, Box G-S121-6, 121 South Main Street, Providence, RI 02912 USA; 4 Center for Gerontology & Healthcare Practice, 1200 Centre Street, Boston, MA 02131 USA

**Keywords:** Pragmatic clinical trial, Pragmatic trial, Effectiveness, Translational research, Implementation science, Model, Framework

## Abstract

**Background:**

Pragmatic randomized, controlled trials (PCTs) test the effectiveness of interventions implemented in routine clinical practice. Because PCT findings are generalizable, this approach is gaining momentum among interventionists and funding agencies seeking to accelerate the testing and adoption of evidence-based strategies to improve care and outcomes. Particular attention is being paid to non-pharmacological interventions, which are often complex and may be difficult to uniformly implement across multiple sites. While many such non-pharmacological interventions have proven efficacious in small trials, most have not been widely adopted. PCTs could accelerate effectiveness testing and adoption, yet there are no established criteria to identify interventions ready for testing in a PCT.

**Methods:**

We convened 30 interventionists and healthcare leaders to identify criteria to assess the readiness of non-pharmacological interventions for PCTs. Based on this discussion, we created a model with multiple domains, qualitative scoring guidelines for each domain, and a graphical summary of readiness assessments. All workshop participants had an opportunity to review and comment on the resulting model; three piloted it with their own interventions. Several other experts also provided input.

**Results:**

The Readiness Assessment for Pragmatic Trials (RAPT) model enables interventionists to assess an intervention’s readiness for PCTs. RAPT includes nine domains: implementation protocol, evidence, risk, feasibility, measurement, cost, acceptability, alignment, and impact. Domains reflect a range of considerations regarding the feasibility of successfully employing PCT methods and the prospect of an intervention’s widespread adoption, if proven effective. Individuals evaluating an intervention are asked to qualitatively assess each domain from low to high readiness. In this report, we provide assessment guidelines and examples of scored interventions.

**Conclusions:**

RAPT is the first model to help interventionists and funders assess the extent to which interventions are ready for PCTs. Scoring efficacious interventions using RAPT can inform research team discussions regarding whether or not to advance an intervention to effectiveness testing using a PCT and how do design that PCTs.

## Background

Pragmatic randomized, controlled trials (PCTs), which test effectiveness under real-world conditions [1], may help to address the need to accelerate the adoption of evidence-based interventions. Because they can be undertaken relatively quickly and result in highly-generalizable findings, PCTs are gaining favor among interventionists and funding agencies. Since 2012, the National Institutes of Health (NIH) Common Fund has been strengthening national capacity to conduct PCTs in healthcare systems through its Health Care System Collaboratory, which captures and publishes best practices from more than a dozen NIH-funded PCTs [2]. More recently, the National Institute on Aging (NIA) and other stakeholders have sought to stimulate PCTs focused specifically on non-pharmacological dementia interventions, [3] recognizing that few efficacious dementia interventions have been replicated.

While a widely-adopted framework, the Pragmatic–Explanatory Continuum Indicator Summary (PRECIS) [4] helps trialists assess the pragmatism of their study design when undertaking PCTs, there are no established criteria to assess efficacious interventions for their readiness for PCTs—and moving forward with a PCT when an intervention is not sufficiently “ready” can have serious consequences, ranging from wasted time and money to false conclusions. To address the need to formally assess interventions’ readiness for PCTs, we convened experts to recommend criteria that researchers should consider in decision-making and trial design. This paper presents the resulting Readiness Assessment for Pragmatic Trials (RAPT) model.

## Methods

As an official activity following a federally-funded national research summit on dementia care [5] the National Institute on Aging sponsored a one-day expert workshop in December 2017. Part of the agenda centered on generating recommendations regarding criteria to assess the readiness of interventions for PCTs.

The workshop and its results, including infrastructure recommendations, are detailed elsewhere [6, 7]. Three of the authors served as chairs (RB, SM, and VM) and two presented and attended (EJ and EM). Participants (*N* = 30; listed in the Acknowledgements) included researchers with expertise relevant to the conduct of PCTs, such as data, regulatory, and ethical issues; interventionists; and healthcare leaders with experience translating evidence-based interventions into practice. Senior NIA staff (*N* = 6) also attended.

After the workshop, we summarized participants’ recommended criteria for determining the characteristics of interventions ready for PCTs [6, 7], and used recommended criteria to define domains to assess interventions for PCT readiness. Inspired by the PRECIS framework, we developed initial guidelines for assessing each domain and a graphical summary to plot domain assessments. We emailed the resulting model to the 30 workshop participants and asked them to review the content and to pilot test an assessment, if possible, with their own intervention.

Finally, we presented an updated draft to approximately 30 additional experts, including interventionists, who attended a Brown and Hebrew SeniorLife seminar in a series focused on the design and execution of pragmatic and cluster-randomized trials. The final model reflects discussion from this seminar.

## Results

**Fig. 1:**
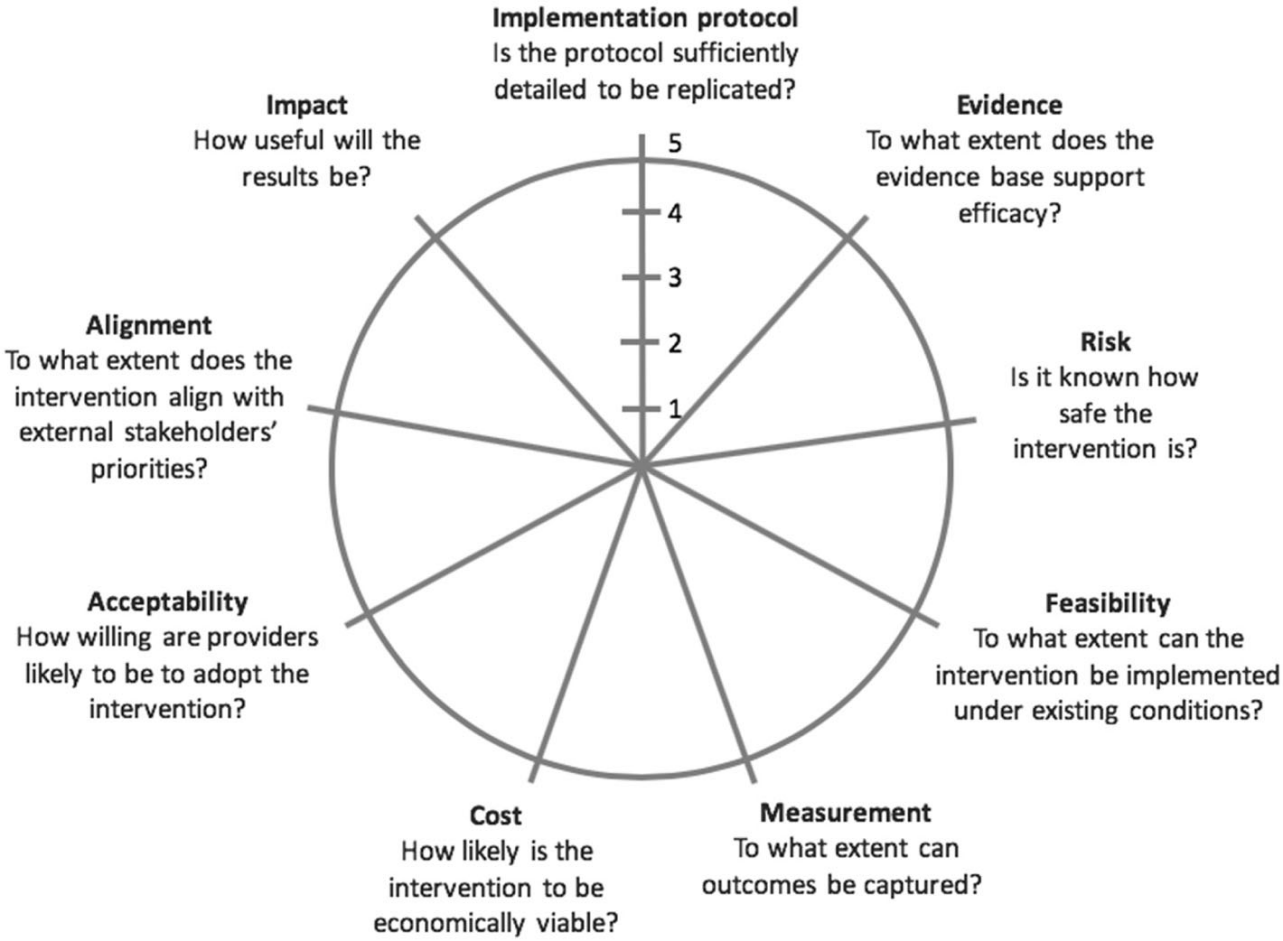
The blank Readiness Assessment for Pragmatic Trials (RAPT) graphical summary “wheel”

We made minor iterative revisions to RAPT based on feedback first from NIA workshop participants and later from Brown and Hebrew SeniorLife seminar attendees. Of the 30 workshop participants, seven provided thorough comments and three pilot tested it with their interventions. Most suggestions involved minor wordsmithing changes, with the exception of shifting the assessment from a numeric to a qualitative scale; we made this final change following the group discussion at the Brown and Hebrew SeniorLife seminar. The resulting model includes nine domains that reflect a range of considerations regarding the feasibility of successfully employing PCT methods to test a non-pharmacologic intervention and the prospect of an intervention’s widespread adoption, if proven effective. The model asks individuals assessing an intervention to score each domain on a spectrum from low to high and enables them to summarize results graphically (Fig. 1). Table 1 provides scoring guidance to consider.

**Table 1:** Readiness Assessment for Pragmatic Trials (RAPT) domains and scoring guidance

Domain	Assessment	Scoring Guidance		
		Low	Medium	High
1. Implementation protocol^a^	Is the protocol sufficiently detailed to be replicated?	There is no protocol.	The protocol provides some documentation, but may be difficult to replicate.	The protocol is well documented and is likely to be replicable.
2. Evidence	To what extent does the evidence base support the intervention’s efficacy?	There are no efficacy studies or the efficacy studies did not use rigorous methods (e.g., a RCT).	A single study using rigorous methods demonstrated efficacy.	Multiple studies using rigorous methods have demonstrated efficacy.
3. Risk	Is it known how safe the intervention is?	The risks (harms and discomforts) are unknown or are known to be more than minimal (e.g., greater than ordinarily encountered in daily life).	The risks are unknown, but are likely minimal.	The risks are known to be minimal.
4. Feasibility	To what extent can the intervention be implemented under existing conditions?	Resources necessary for implementation (e.g., staff, infrastructure, payment) are absent or insufficient.	Minor modifications to existing resources would enable implementation.	Implementation is possible with existing resources.
5. Measurement	To what extent can the intervention’s outcomes be captured?^a^	Outcomes cannot be captured without major modifications to systems (e.g., clinical assessments, documentation, or electronic health records) or increases in staff time.	Outcomes can be captured with minor modifications to systems or increases in staff time.	Outcomes are already routinely captured.
6. Cost	How likely is the intervention to be economically viable?	Cost-benefit/cost-effectiveness analysis has not been completed (formally or informally) and it is unknown whether benefits outweigh costs.	Cost-benefit/cost-effectiveness analysis has not been completed, but benefits are likely to outweigh costs.	Cost-benefit/cost-effectiveness analysis demonstrates benefits outweigh costs.
7. Acceptability	How willing are providers likely to be to adopt the intervention?	Acceptability is unknown or staff are unlikely to believe the intervention is feasible or needed.	Acceptability is unknown, but staff are likely to believe the intervention is feasible or needed.	Acceptability is known and staff believe the intervention is feasible and needed.
8. Alignment	To what extent does the intervention align with external stakeholders’ priorities?	Stakeholders (policymakers, payors, advocates, and others) do not believe the intervention addresses a current or anticipated priority.	Some stakeholders believe the intervention addresses a priority.	Most or all stakeholders believe the intervention addresses a priority.
9. Impact	How useful will the intervention’s results be?	Providers and stakeholders (policymakers, payors, advocates, and others) are unlikely to believe that the outcomes are useful (e.g., to inform clinical care or policy).	Some providers or stakeholders are likely to believe the outcomes are useful.	Most or all providers and stakeholders are likely to believe the outcomes are useful.

^a^Refers to the operations manual or implementation guide for the intervention’s deployment, not the Institutional Review Board submission

*PCT* Pragmatic randomized, controlled trial, *RAPT* Readiness Assessment for Pragmatic Trials, *RCT* Randomized, controlled trial

### Implementation protocol

Is the intervention protocol sufficiently detailed to be replicated? In this context, protocol refers to an operations manual or implementation guide, not the Institutional Review Board submission. To be ready for a PCT, an intervention should have a well-documented protocol. Ideally, an intervention’s outcomes should be measurable and captured using existing data sources or systems, so that the intervention – if proven effective – can be readily adopted on a broad scale. Although it may be possible to make minor revisions to existing systems, such as electronic health records, major revisions and any data collection that would burden staff will affect widespread adoption.

### Evidence

To what extent does the evidence base support the intervention’s efficacy? Ideally, there should be an evidence base establishing efficacy using rigorous methods, such as randomized, controlled trials (RCTs). Efficacy studies may also have demonstrated which intervention components, alone or in combination, are associated with improvement. An intervention with mixed findings or less robust evidence may not be sufficiently efficacious for a PCT. Furthermore, overwhelming evidence for efficacy doesn’t necessarily translate to overwhelming evidence for effectiveness. While an intervention may work in a controlled setting, it is vital to test the intervention in a real-world context (such as a PCT).

### Risk

Is it known how safe the intervention is? PCTs often involve providers implementing interventions as a new standard of care with all patients. An intervention should therefore be of minimal risk, with careful consideration given to potential adverse events and unintended consequences. This is particularly true when an intervention targets people who are vulnerable, such as those with ADRD or residing in nursing homes and other residential care settings.

### Feasibility

To what extent can the intervention be implemented under existing conditions? An intervention should be feasible for providers and healthcare systems to implement as part of routine clinical care, with existing staff, infrastructure, and other resources, such as reimbursement models. If resources are absent or insufficient, this could affect implementation both in the PCT and when the intervention is broadly disseminated.

### Measurement

To what extent can outcomes be captured during the conduct of the PCT? Ideally, an intervention’s outcomes should be measurable and captured using existing data sources or systems, to ensure that the PCT can be implemented and evaluated without resource-intensive primary data collection. Existing data systems may also be useful for ongoing audit and feedback to drive the intervention’s adoption and spread. Although it may be possible to make minor revisions to existing systems, such as electronic health records, major revisions and any data collection that would burden staff will affect widespread adoption.

### Cost

How likely is the intervention to be economically viable? The benefits of an intervention should outweigh its costs, so that there is a realistic business case for providers’ implementation or reimbursement. Without making the business case, providers are unlikely to widely adopt an intervention, even if it demonstrates effectiveness in a PCT.

### Acceptability

How willing are providers likely to be to adopt the intervention? Leadership and frontline staff should believe that an intervention is feasible in their unique environment and workflow and addresses a need. While providers’ perceptions of acceptability may be driven, in part, by evidence demonstrating an intervention’s impact, some baseline understanding of their willingness to adopt the intervention during effectiveness testing will help when determining how likely the intervention is to be implemented fully (with fidelity) or once proven effective.

### Alignment

To what extent does the intervention align with external stakeholders’ priorities? While acceptability focuses on providers, alignment focuses on external stakeholders: policymakers, payors, patients and their families, advocates, and others. An intervention should address a current or anticipated priority for most or all external stakeholders. For example, it may be a topic included in state or national policy, payment, or programs. Without alignment, the intervention is unlikely to be widely adopted.

### Impact

How useful will the results be? As with alignment, this domain relates to the likelihood of the intervention being widely adopted; it differs in that it focuses on the results of the PCT vs. the need for the intervention itself. Providers and stakeholders (policymakers, payors, advocates, and others) should believe that the outcomes will be useful to inform clinical care and policy. An intervention is unlikely to be widely adopted if results are not likely to be perceived as useful.

### Scored examples

**Fig. 2:**
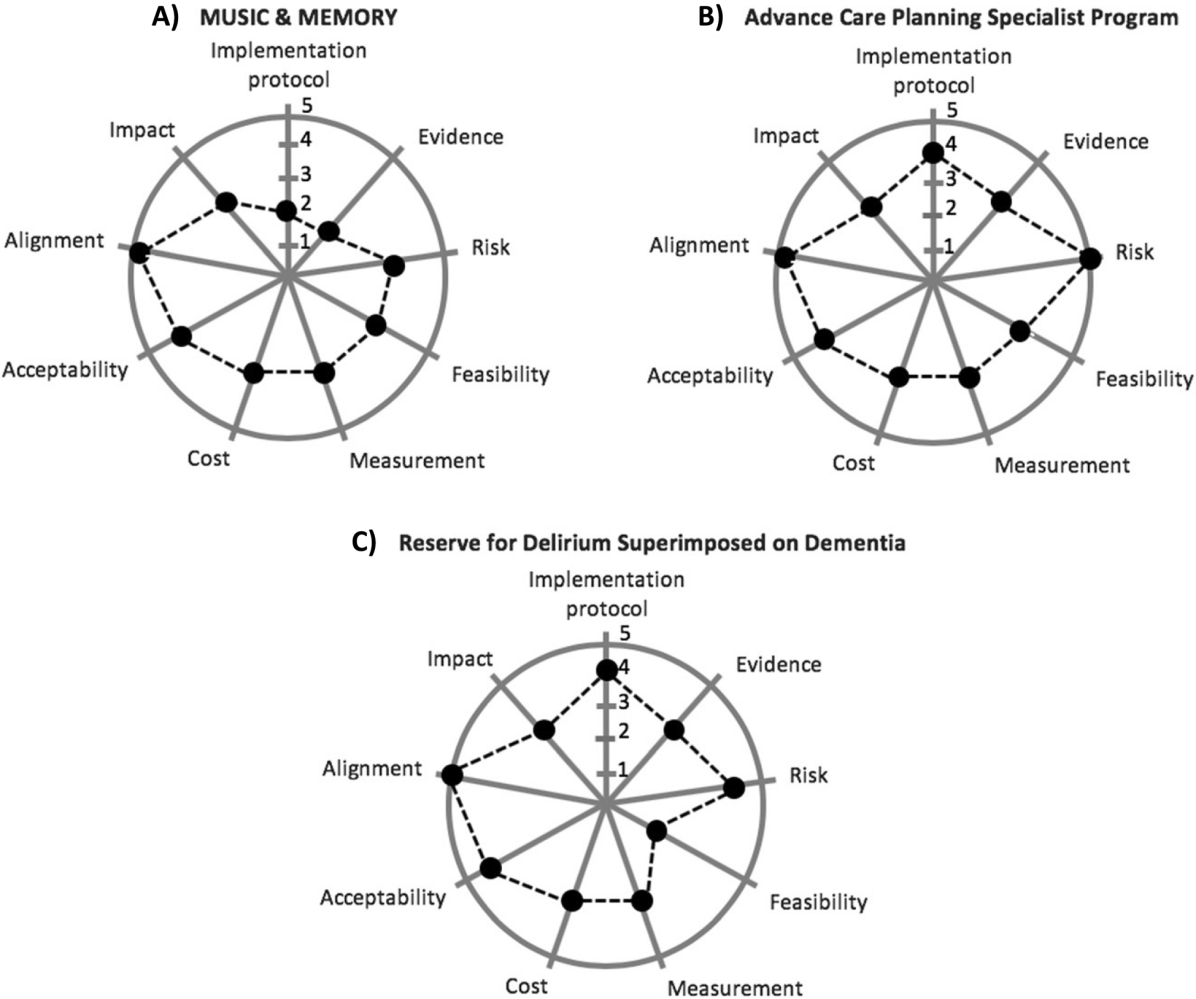
Examples of three scored interventions

Figure 2 presents graphical summaries of RAPT assessments for the three non-pharmacological dementia interventions that serve as examples: A) the MUSIC & MEMORY individualized music program for nursing home residents with dementia, B) Advance Care Planning Specialist Program, and C) Reserve for Delirium Superimposed on Dementia.

MUSIC & MEMORY (A) involves providing individualized music to persons with dementia using personalized music devices [8] and became popular among nursing homes in part because of a widely-viewed documentary, *Alive Inside*, showcasing the intervention [9]. It scores highest on domains related to alignment and acceptability, because it addresses national priorities related to improving dementia care in nursing homes and is already widely adopted by providers familiar with anecdotal results and seeking strategies to improve care for residents with ADRD. It scores lowest on domains related to the intervention’s evidence and implementation protocol, because it has not been prospectively evaluated using rigorous methods and existing training materials, while numerous, stop shy of a detailed protocol. Several of the authors (RB, EM, and VM) are currently undertaking a PCT of MUSIC & MEMORY that included an initial phase designed to address these weaknesses, focused on developing a protocol and pilot testing evaluation strategies [10].

The Advance Care Planning (ACP) Specialist Program (B) is a nursing home intervention that involves providing standardized staff education and strengthening facilities’ procedures, to enable systematic advance care planning facilitation by existing staff [11, 12]. The interventionists are currently implementing a PCT applying the intervention to nursing home residents with ADRD [13]. Because it aims to strengthen a process that is already occurring as part of standard practice, the ACP Specialist Program is low risk and scores high on that domain. It also scores high for alignment, because nursing home staff widely recognize advance care planning as valuable, but challenging, and welcome assistance improving the process. In contrast, medium scores for several domains, such as feasibility and measurement, reflect the fact that the intervention requires nursing home leaders to review and revise existing policies, explicitly commit staff time (which may require shifting commitments), and modify electronic documentation to capture advance care planning.

Reserve for Delirium Superimposed on Dementia (DSD) (C) is an intervention that involves using cognitive stimulation to improve outcomes among persons with dementia receiving post-acute care. While an RCT comparing Reserve for DSD to usual care found no differences in the duration and severity of delirium, it did find better executive function (an important cognitive domain affected by delirium) and a shorter post-acute care length of stay in the intervention group vs. a control group [14]. The intervention scores highest for alignment; most stakeholders believe delirium is a significant clinical problem for persons with dementia because of its effects on health and cost outcomes. It also scores high for protocol, because the RCT involved developing an interventional manual, producing a video on implementing the intervention, and publishing the protocol [15]. The lowest score pertains to feasibility, since the RCT protocol involved using research staff to implement the intervention.

## Discussion

RAPT is the first model to help interventionists and funding agencies, among others, assess the extent to which interventions are ready for effectiveness testing in routine clinical practice using PCT methods. Assessing an intervention using RAPT may inform discussion and decisions about whether to proceed with a PCT and, in conjunction with PRECIS, how best to design that PCT, including any preliminary work necessary to complete prior to proceeding.

This model arose from discussion at a NIA-funded workshop that focused on non-pharmacological dementia interventions. A federally-funded research summit in 2017 concluded that improving care for persons with dementia is an urgent public health challenge requiring high-quality evidence, [5] yet few efficacious studies have been consistently replicated [16]. The NIA workshop, which followed the summit, therefore focused on interventions and PCTs targeting this population. Nonetheless, we believe RAPT is broadly applicable to interventions across all clinical fields: none of the nine domains are specific to dementia and many of those who helped to vet the model were interventionists whose research does not center on dementia.

An intervention’s RAPT assessment can be plotted on a wheel, allowing interventionists to graphically summarize all nine domains and to quickly identify any domains requiring particular consideration. Some RAPT domains may be unknown prior to a PCT (e.g., risk or evidence) and will subsequently receive a low assessment; however, this alone should not prevent researchers from moving forward with a PCT. We do not propose any specific weighting or any thresholds for “passing” the overall assessment and likewise do not recommend directly comparing interventions’ assessments to one another. Rather, we suggest that each intervention’s readiness for a PCT be considered based on its individual strengths and weaknesses, with results used to inform the research team’s discussion about whether or not to advance an intervention to a PCT and—together with PRECIS—how to design that PCT. In other words, a low assessment may not preclude conducting a PCT, but could give insight into considerations that improve the PCT’s design and conduct.

To increase reliability, we also recommend having multiple people independently score an intervention and then compare results to resolve any discrepancies and inform discussion. While RAPT includes detailed scoring guidance, some domains are more subjective than others. For example, it is relatively straightforward to ascertain whether or not an intervention has a detailed implementation protocol, such as an operations manual (protocol domain). However, assessing providers’ beliefs about whether an intervention is feasible and needed (acceptability domain) may prove difficult for interventionists, unless the research team includes clinicians or others intimately familiar with the day-to-day realities of the targeted healthcare setting. Identifying ways to incorporate providers’ thoughts may be necessary; just as important as incorporating the view of patients and families into the initial development of interventions.

In developing RAPT, we applied it to our own intervention, Music & Memory, post-hoc. This allowed us to recognize weaknesses—particularly around feasibility—that would have been helpful to discuss in detail when designing the large PCT that we are now conducting in nursing homes across the country. Thankfully, our trial did include a pilot phase that enabled us to identify and address critical issues before proceeding with our full-scale PCT. The pilot was intended to include transforming training materials into a detailed implementation protocol, addressing an area in which the intervention did score low in our example. It ultimately also addressed issues we didn’t adequately foresee, particularly around the feasibility of implementing a seemingly “simple” intervention in low-resource nursing homes that lack quality improvement infrastructure, face frequent leadership turnover, and include numerous competing priorities. We incorporated pilot findings into the implementation protocol and trial design.

## Conclusions

RAPT was developed based on expert recommendations and is the first model to help interventionists and funding agencies determine the extent to which interventions are ready for PCTs. Evaluating efficacious interventions using RAPT can inform research team discussion regarding whether or not to advance an intervention to effectiveness testing using a PCT and how do design that PCT.

## Data Availability

Not applicable.

## Abbreviations

ACP: Advance Care Planning
ADRD: Alzheimer’s disease and related dementias
DSD: Delirium Superimposed on Dementia
NIA: National Institute on Aging
PCT: Pragmatic randomized, controlled trial
RAPT: Readiness Assessment for Pragmatic Trials
RCT: Randomized, controlled trial
